# Patient-reported outcome measures in patients with peripheral arterial disease: a systematic review of psychometric properties

**DOI:** 10.1186/s12955-016-0563-y

**Published:** 2016-11-24

**Authors:** Edith Poku, Rosie Duncan, Anju Keetharuth, Munira Essat, Patrick Phillips, Helen Buckley Woods, Simon Palfreyman, Georgina Jones, Eva Kaltenthaler, Jonathan Michaels

**Affiliations:** 1 School of Health and Related Research, University of Sheffield, Sheffield, S1 4DA UK; 2 University of Alberta, 116 St & 85 Ave, Edmonton, T6G 2R3 AB Canada; 3 Leeds Beckett University, School of Social Sciences, City Campus, Leeds, LS1 3HE UK

**Keywords:** Patient-reported outcome measures, Peripheral arterial disease, Psychometric, Validation, Systematic review

## Abstract

**Background:**

Peripheral arterial disease (PAD) is generally associated with considerable morbidity and reduced quality of life. Patient-reported outcome measures (PROMs) provide important information about the burden of disease and impact of treatment in affected patients.

**Objectives:**

The objective of the review was to identify and appraise studies reporting the psychometric evaluation of PROMs administered to a specified population of patients with PAD with a view to recommending suitable PROMs.

**Methods:**

A systematic review of peer-reviewed English language articles was undertaken to identify primary studies reporting psychometric properties of PROMs in English-speaking patients with various stages of PAD. Comprehensive searches were completed up until January 2015. Study selection, data extraction and quality assessment were undertaken independently by at least two researchers. Findings were presented as tabular and narrative summaries based on accepted guidance.

**Results:**

Psychometric evaluation of 6 generic and 7 condition-specific PROMs reported in 14 studies contributed data to the review. The frequently reported measure was the SF-36 (*n* = 11 studies); others included the Walking Impairment Questionnaire (*n* = 8 studies), EQ-5D (*n* = 5 studies) and the Vascular Quality of Life Questionnaire (*n* = 3 studies). Studies included a diverse PAD population and varied in methodology, including approach to validation of PROMs.

**Conclusions:**

Various PROMs have been validated in patients with PAD but no study provided evidence of a full psychometric evaluation in the patient population. Careful selection is required to identify reliable and valid PROMs to use in clinical and research settings.

**Electronic supplementary material:**

The online version of this article (doi:10.1186/s12955-016-0563-y) contains supplementary material, which is available to authorized users.

## Background

Patient-reported outcome measures (PROMs) reflect patients’ perspectives on their health status, functioning and quality of life (QoL) [1] and are also useful for informing clinical and healthcare decision-making [2]. Since April 2009, the National Health Service (NHS) in England requires patients undergoing surgery to provide PROMs data before and after treatment. The current PROMs programme covers patients undergoing varicose vein, groin hernia, knee replacement and hip replacement surgery [3]. Presently, PROMs are not routinely collected for patients with peripheral arterial disease (PAD), a condition associated with substantial disability, morbidity and mortality [4]. PAD is caused by widespread atherosclerosis of the lower limbs and may be asymptomatic in the early stages. An initial common presentation of PAD is atypical leg pain. Pain may occur in a specific group of muscles in the lower limb during effort (this is referred to as intermittent claudication). Severe stages of PAD present as rest pain in the legs, leg ulcers or gangrene—collectively known as critical limb ischaemia (CLI). The mainstay of treatment is to improve symptoms, delay disease progression, prevent tissue loss and modify risk factors [4, 5].

Validation studies provide valuable evidence for selecting appropriate PROMs for use in clinical and research settings. In this review, the term validation study refers to a study reporting the evaluation of one or more measurement properties of a PROM—including its validity (the degree to which the instrument measures what it is supposed to measure); reliability (the degree to which measures are reproducible and consistent over time in patients with a stable condition); responsiveness (the degree to which the instrument detects meaningful change over time) and acceptability (the degree to which the instrument is acceptable to the patient). A suitable PROM must demonstrate its validity, reliability, responsiveness and appropriateness in a relevant patient population [6]. Confirmation of these psychometric properties must be obtained from sources (i.e. context of study, patient factors and study characteristics) similar to those in which the PROMs will be applied [6].

A better understanding of the psychometric properties of PROMs obtained from English-speaking patients with PAD will help to select an appropriate tool for patients managed within the NHS. Therefore, this study sought to (1) identify English language publications reporting the psychometric evaluation of PROMs in patients with PAD, (2) critically appraise eligible studies, and (3) examine the psychometric properties of identified PROMs to inform the development of a valid and reliable instrument to incorporate into an electronic personal assessment questionnaire (ePAQ) as part of a project to inform the reconfiguration of vascular services in the UK..

## Methods

A systematic review of peer-reviewed English language articles was undertaken according to recommendations of the Preferred Reporting Items for Systematic Reviews and Meta-Analysis (PRISMA) group [7], the Oxford system and the Consensus-based Standards for the selection of health Measurement Instruments (COSMIN) group [8, 9] with the aim to identify validation studies in a well-defined population of English-speaking patients with symptomatic PAD. The study’s protocol is available on request from the authors.

### Literature searches

Comprehensive searches using a two-staged approach were conducted in Medline and Medline in Process, EMBASE, the Cochrane Library, CINAHL, PsycINFO and Web of Science from date of inception up to August 2013 (Search 1) and up to February 2014 (Search 2). Updated searches were conducted in Medline and Medline in Process in January 2015. Search 1 sought to identify studies reporting PROMs in patients with PAD while Search 2 aimed to identify studies reporting the development and/or validation of relevant PROMs. Relevant PROM terms were identified from scoping searches, discussions with experts and previous research relating to relevant outcome measures. Search terms in the search 1 strategy included free text terms and Medical Subject Heading (MeSH) terms related to: (1) PAD; (2) known generic PROMs and (3) known condition-specific PROMs. Additional PROMs were identified following examination of titles and abstracts of records retrieved from Search 1. All potentially relevant articles were also coded at this stage. The search 2 strategy comprised of all terms used in the search 1, together with (1) additional PROM terms identified from sifting retrieved records and (2) a methodological search filter for locating studies reporting measurement properties. Search strategies were adapted for searching within different databases. Search strategies used in Medline are available as Additional file 1.

Further searches were conducted in the PROMs Bibliography (Oxford University) and the Patient-Reported Outcome and Quality of Life Instruments database (PROQOLID) [10]. References of identified systematic reviews and included studies were examined for potentially eligible studies. All retrieved records were transferred and managed within a single reference management database.

### Study selection

Study selection was undertaken by one reviewer from a pool of 4 reviewers (EP, ME, PP, RD) and checked by a second reviewer. Eligibility criteria are summarised in Table 1. Disagreements were resolved by discussion and referred to a third reviewer, when needed. After excluding duplicates and records which did not appear to be relevant by examination of titles and abstracts, all full-text articles of potentially relevant articles were obtained for detailed review.

**Table 1:** Criteria for considering eligibility of studies for inclusion in the review

	Inclusion criteria	Exclusion criteria
Population	Defined population of English-speaking participants aged 18 years (adults) with PAD^a^ Patients with rest pain; claudication; vascular spasms; ischaemic ulceration; amputation; necrosis or gangrene of the limb due to PAD	Undefined population or Non-English speaking adults with PAD Patients with rest pain; claudication; vascular spasms; ischaemic ulceration; amputation; necrosis or gangrene of the limb due to any cause other than PAD
Interventions	No intervention or any intervention indicated for PAD	Intervention, not intended for the management of PAD
Outcomes	Original version of PROMs in English including • generic or preference-based measures e.g. EQ-5D, SF-6D, SF-36; • directly elicited preference-based measures e.g. time-trade-off (TTO), standard gamble (SG) utility values; condition-specific outcome measures; • functional outcome measures	Original version of PROMs in English including • Outcome measures of patient satisfaction or experience • Outcome measures obtained from proxies, carers or health providers Non-English versions of PROMs English translations of non-English PROMs
Study type	Validation studies of a relevant PROM addressing • Validity; • Reliability; • Responsiveness or acceptability Publication in English	Studies of linguistic validation of PROMs Review articles, letters, commentaries, abstracts Non-English publications Unpublished studies

*Abbreviations*: *EQ-5D* EuroQoL-5D, *PAD* peripheral arterial disease, *PROM* patient-reported outcome measure, *SF-6D* 6-item shortened version of SF-36, *SF-36* Medical Outcomes Study 36-item short form health survey, *SG* standard gamble, *TTO* time trade-off

^a^Other descriptions considered included peripheral vascular disease; peripheral obliterative arteriopathy; peripheral arterial occlusive disease

Studies including English-speaking patients with a diagnosis of PAD were included in the review. Proficiency in English was indicated or assumed if studies were conducted in countries where English is an official language and/or reported that 80% or more of participants were English speakers. Studies published in English but reporting outcomes obtained from translated instruments, i.e. non-English translations of relevant PROM instruments or English versions of non-English PROMs were excluded. This was considered as an acceptable approach to overcome the uncertainty due to language validation and cross-cultural adaptation of PROMs [11].

### Data extraction

Data extraction was completed by one author (either EP, ME, PP, RD or AK) and checked by another author. All disagreements were discussed and resolved by consensus. Data were abstracted into a piloted standardised form and comprised patient characteristics, study characteristics, names, domains, items and reported psychometric evaluations of identified PROMs.

### Quality assessment

The methodological quality of studies was assessed using the COSMIN checklist [12]. This checklist comprises of 114 items organised as 12 boxes related to the following measurement properties: validity (including structural validity, content validity, criterion validity and cross-cultural validity), internal consistency, reliability, measurement error, responsiveness and hypothesis-testing. A 4-point rating scale (excellent, good, fair or poor) was applied with the overall methodological quality scores presented using a “worst score counts method” per box [13]. The COSMIN checklist also covers interpretability and generalisability which were assessed but not scored.

Due to the lack of consensus on how to appraise PROMs, study-specific criteria were adapted from various sources [2, 8, 14–17] as outlined in Table 2 and used for the assessment of psychometric performance of identified PROMs.

**Table 2:** Appraisal criteria for assessing the psychometric properties of patient reported outcome measures

Domain	Criteria
Test re-test reliability	*Reliability is the ability of a measure to reproduce the same value on two separate administrations when there has been no change in health.* The intra-class correlation/ weighted kappa score should be ≥ 0.70 for group comparisons and ≥ 0.90 if scores are going to be used for decisions about an individual based on their score [2]. The mean difference (paired *t* test or Wilcoxon signed-rank test) between time point 1 (T_1_) and time point 2 (T_2_) and the 95% CI should also be reported.
Internal consistency	*Internal consistency is an assessment of whether the items are measuring the same thing.* A Cronbach’s alpha score of ≥ 0.70 is considered good and it should not exceed ≥0.92 for group comparisons as this is taken to indicate that items in the scale could be redundant. Item total correlations should be ≥0.20 [14].
Content validity	*Content validity measures the extent to which the items reflect the domains of interest in a way that is clear.* To achieve good content validity, there must be evidence that the instrument has been developed by consulting patients, experts as well as undertaking a literature review. Patients should be involved in the development stage and item generation. The opinion of patient representatives should be sought on the constructed scale [2, 14, 16].
Construct validity	*Construct validity assesses how well an instrument measures what it was intended to measure.* A correlation coefficient of ≥0.60 is considered as strong evidence of construct validity. Authors should make specific directional hypotheses and estimate the strength of correlation before testing [2, 14, 15].
Criterion validity	*Criterion validity assesses the degree of empirical association of the PROM with external criteria or other measures.* A good argument should be made as to why an instrument is a gold standard and correlation with the gold standard should be ≥ 0.70 [15].
Responsiveness	*Responsiveness assesses the ability of the PROM to detect changes when changes are expected.* Available methods to measure responsiveness include t-tests, effect size, standardised response means or responsiveness statistics, Guyatts’ responsiveness index. Standardised effects sizes and SRMs of less than 0.2 are considered small, 0.5 moderate, and 0.8 [17]. There should be statistically significant changes in score of an expected magnitude [8].
Floor-ceiling effects	A floor or celling effect is considered if 15% of respondents are achieving the lowest or the highest score on the instrument, respectively [15].
Acceptability	Acceptability is reflected by the completeness of the data supplied. 80% or more of the data should be complete [16].

### Data synthesis and analysis

Tabular and narrative syntheses of study characteristics were undertaken. A summary of psychometric criteria was completed based on the Oxford system and the COSMIN group system [8, 9]. The following combined rating scales were allocated: (0) for not reported; (−) for evidence not in favour; (+/−) for conflicting evidence; (?) for questionable methodology and (+) for evidence in favour.

## Results

**Fig. 1:**
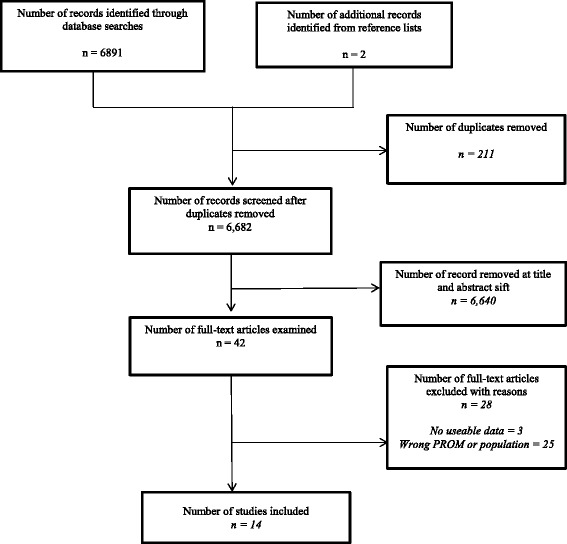
Flow diagram of study selection here

Of the 6893 records retrieved from searches, 14 studies with data for 13 PROMs were found to be eligible to be included in this review as shown in Fig. 1. Twenty-eight full-text articles were excluded because they reported outcomes using *‘non-eligible’* PROMs (i.e. English translations of non-English PROMs and non-English versions of relevant PROMs), included study populations for whom outcomes were not clearly reported or presented no data on psychometric evaluations.

### Study characteristics

Table 3 provides a summary of study characteristics. Studies were conducted in Australia [18], UK [19–25] and the USA [26–31]. All studies were conducted as prospective observational studies. Missing information relating to study setting [22, 30, 31], diagnostic criteria of participants [18, 24, 26, 28, 30, 31] and schedule for assessment of PROMs was noted.

**Table 3:** Table of characteristics of included studies

Author year, country	Reported PROM (s)	Clinical presentation (Sample size) (Ankle brachial index cut-off)	Age (years)	Gender (% males)	Timing of PROM (s) assessment	Concomitant treatment
Chetter 1997, UK [19]	EQ-5D SF-36 NHP	Peripheral arterial disease (*n* = 235)	68^◊^	61	Baseline, week 1	NR
		(NR)				
Chong 2002, UK [20]	EQ-5D ICQ SF-36 WIQ	Intermittent claudication (*n* = 124)	71^◊^	61	Baseline, week 2, month 3	conservative medical treatment; percutaneous transluminal angioplasty
		(≤0.9)				
Coyne 2003, USA [26]	EQ-5D PAD symptom scale, SF-36, WIQ (self-administered and telephone-administered)	Peripheral arterial disease (*n* = 60)	67	78	Baseline, day 4, 7, 14 and 28	NR
		(<0.9, at rest)				
Gulati 2009, UK [21]	SF-36 SF-8	Peripheral arterial disease (*n* = 193)	66^◊^	70	Baseline; at week 2	NR
		(NR)				
Izquierdo-Porrera 2005, USA [27]	SF-36 WIQ	Intermittent claudication	71	91	Baseline, at week 1	exercise rehabilitation
		(*n* = 80) (<0.97, at rest; < 0.85, 0.85 during recovery from exercise))				
Mazari 2010, UK [25]	EQ-5D SF-6D VascuQoL	Intermittent claudication (*n* = 178)	70^◊^	60	Baseline, at month 1, 3, 6, 12	transluminal angioplasty, supervised exercise program, or combined treatment
McDermott 1998, UK [28]	WIQ	Intermittent claudication (*n* = 146^a^)	71.4	57	Baseline	NR
		(≤0.9, at rest)				
Mehta 2006^b^, UK [22]	EQ-5D SF-36 SIPic VascuQol	Intermittent claudication (*n* =70)	70^◊^	54	Baseline, at month 6	Percutaneous transluminal angioplasty (*n* =47); Conservative medical therapy (*n* = 23).
		(NR)				
Morgan 2001, UK [23]	SF-36 VascuQol	Peripheral arterial disease (*n* = 39)	67^◊^	62	Baseline, at week 4	general advice, medical treatment, angioplasty (*n* = 4); bypass surgery (NR)
		(NR)				
Regensteiner 1990, USA [29]	WIQ	Intermittent claudication (*n* = 26)	59 (exercise group); 64 (surgery group) 61 (control group)	NR	Baseline, at week 1, 6 and 12	supervised exercise (*n* = 10); bypass surgery (*n* = 7)
		(<0.90, at rest; < 0.85, after exercise)				
Smith 2007, Australia [18]	SF-36 AUSVIQOL	Intermittent claudication (*n* = 71)	72.8	68	Baseline, at month 1	NR
		(NR)				
Spertus 2004, USA [30]	WIQ PAQ SF-36	Peripheral arterial disease (*n* = 44)	68	55	Baseline; at week 2 and 8	Peripheral revascularization
		(NR)				
Tew 2013^c^, UK [24]	WIQ	Intermittent claudication	65	81	Baseline, within days 7 to 10 of first visit	NR
		(*n* = 37)				
		(≤0.9, at rest)				
Treat-Johnson 2012, USA [31]	PADQOL POMS SF-36 WIQ	Peripheral arterial disease (*n* = 295)	67.9	75	Baseline; follow-up (not specified)	NR
		(NR)				

*Abbreviations*: *AUSVIQUOL* Australian Vascular Quality of Life Index, *EQ-5D* EuroQoL-5D, *IC* intermittent claudication, *ICQ* Intermittent Claudication Questionnaire, *M* male, *NR* not reported, *NHP* Nottingham Health Profile, *PAD* peripheral arterial disease, *PADQOL* PAD Quality of Life Questionnaire, *PAQ* Peripheral Artery Questionnaire, *POMS* Profile of Mood States, *SF-36* Medical Outcomes Study 36-item Short Form, *SF-6D* 6-item shortened version of SF-36, *SF-8* 8-item shortened version of SF-36, *SIPic* Sickness Impact Profile–Intermittent Claudication, *VascuQoL* King’s College Hospital’s Vascular Quality of Life instrument, *WIQ* Walking Impairment Questionnaire

^◊^Indicates median age, other values relate to reported mean ages

^a^Data presented for sub-group of study population with PAD only

^b^Study also reported the validation of English version of the Claudication Scale (CLAU-S)

^c^Study also reported the validation of English version of the Estimation of Ambulatory Capacity by History-Questionnaire (EACH-Q)

### Participants’ characteristics

Data were available for 1594 patients presenting with symptomatic PAD. Sample sizes ranged from 26 to 295 patients with more than 50% of included studies reporting study populations of less than 100 participants. Overall, men made up between 54 [22] and 91% [27] of study populations. Diagnostic criteria and management strategies varied across studies. Included studies fell into 2 broad categories based on diagnosis of patients: studies with (i) patients with IC only [18, 20, 22, 24, 27, 29] or (ii) patients with different degrees of severity of PAD [19, 21, 23, 26, 28, 30, 31].

### Psychometric data

Data relating to the psychometric evaluation of 6 generic PROMs and 7 condition-specific PROMs in patients with PAD were available. The most frequently assessed generic questionnaires were the SF-36 [18–23, 26, 27, 30, 31] and the EQ-5D [19, 20, 22, 26]. The King’s College Hospital Vascular Quality of Life Questionnaire (VascuQoL) [22, 23, 25] and the Walking Impairment Questionnaire (WIQ) [20, 24, 26–31] were the commonly reported condition-specific measures. Two studies reported the evaluation of the Claudication Scale (CLAU-S) and the Estimation of Ambulatory Capacity by History Questionnaire (EACH-Q) which were originally developed in France [24] and Germany [22], respectively, alongside relevant PROMs. Information relating to the CLAU-S and EACH-Q was excluded in this review.

Information about the development of the WIQ [29]; Intermittent Claudication Questionnaire (ICQ) [20]; VascuQoL [23]; Peripheral Artery Questionnaire (PAQ) [30] and the PAD Quality of Life Questionnaire (PADQOL) [31] was found in 5 studies. Limited information about the development of the WIQ was noted [29], however for the remaining instruments studies reported methods consistent with recommended standards [11, 32]. Items, domains, response options and scoring of identified PROMs are presented in Table 4.

**Table 4:** Table of items, domains, response options, scoring and administration of included outcome measures

Instrument (number of items)	Domains (number of levels)	Measure: Response options	Scoring	Mode of administration (reported completion time, min)
Generic PROMs				
SF-36 (36) [18–23, 25–27, 30, 31]	Vitality (4), physical functioning (10), bodily pain (2), general health perceptions (5), physical role functioning (4), emotional role functioning (3), social role functioning (2), mental health (5)	Likert scale: 2 to 5	Each dimension is transformed to give a score of 0 to 100. Lower scores indicating greater disability	Self-completed (11 min)
EQ-5D (6) [19, 20, 22, 25, 26]	Mobility (1), self-care (1), usual activities (1), pain/discomfort (1), and anxiety/depression (1); VAS	Likert scale: 3; VAS	Preference based, values range from 0 indicating death to 1 representing perfect health	Self-completed
SF-6D [25]	Physical functioning (1), role limitation (1), social functioning (1), pain (1), mental health (1), and vitality (1)	Likert scale: 4 to 6	Preference based 0 = dead to 1 = perfect health	Self-completed
SF-8 (8) [21]	Vitality (1), physical functioning (1), bodily pain (1), general health perceptions (1), physical role functioning (1), emotional role functioning (1), social role functioning (1), mental health (1)	Likert scale: 5	Each dimension is transformed to give a score of 0–100. Lower scores indicating greater disability	Self-completed (2.5 min)
NHP (38) [19]	Physical mobility (8), pain (8), sleep (5), energy (3), emotional reactions (9), and social isolation (5)	Dichotomous	0 (no health problems) to 100 (all the health problems)	Self-completed
POMS (65) [31]	NR	Likert scale: 5	NR	Self-completed
Condition-specific PROMs				
AUSVIQUOL (10) [18]	General health perceptions (3), function, mobility and pain (5), psychosocial aspects (2)	Likert scale: 5	Reponses are given points from 10 to 0 for each answer, these are summed to give a quality of life score ranging from 0 (poor) to 100 (excellent)	Interviewer or self-completed (3.27 min)
ICQ (16) [20]	Health related quality of life (16)	Likert scale: 5	Summing scores and transforming to a 0–100 scale	Self-completed (3.7 min)
PAQ (20) [30]	Physical limitation (7), symptoms (4), quality of life (3), social function (3), treatment satisfaction (3)	Likert scale: 5	0–100 (lower scores indicating worse performance)	Self-completed
PADQOL (38) [31]	Social relationships and interactions (9), self-concept and feelings (7), symptoms and limitations in physical functioning (8), fear and uncertainty (4), positive adaptation (7)	Likert scale :5	Summed and transformed score 0 to 100%	Self-completed (5 to10 min)
SIPic (12) [22]	Sickness related behaviour (12)	Number of items endorsed	0 (best quality of life) to 12 (worst quality of life)	Self-completed
WIQ (14) [20, 24, 26–31]	Symptom severity (8) Walking distance (7), walking speed (4), stair climbing (3)	Likert scale: 5	0 (unable to do) to 4 (no difficulty)	Self-completed (6 min)
VascuQol (25) [22, 23, 25]	Pain (4), activity (8), emotional (7), symptoms (4), and social (2)	Likert scale: 7	1 (the worst) to7 (the best possible)	Self-completed

Abbreviations: AUSVIQUOL Australian Vascular Quality of Life Index, EQ-5D EuroQol, ICQ Intermittent Claudication Questionnaire, NR not reported, NHP Nottingham Health Profile, PAD peripheral arterial disease, PADQOL PAD Quality of Life Questionnaire, PAQ Peripheral Artery Questionnaire, POMS Profile of Mood States, SF-36 Medical Outcomes Study 36-item Short Form, SF-6D 6-item shortened version of SF-36, SF-8 8-item shortened version of SF-36, SIPic Sickness Impact Profile–Intermittent Claudication, VAS visual analogue scale, VascuQoL King’s College Hospital’s Vascular Quality of Life instrument, WIQ Walking Impairment Questionnaire

In relation to the COSMIN checklist, the methodological quality was assessed by totalling the number of boxes that have been scored from poor to excellent. Of 36.8% of the included studies (*n* = 42 boxes) was rated as poor, 40.3% (*n* = 46 boxes) as fair; 21.9% (*n* = 25 boxes) as good and 0.9% (*n* = 1 boxes) as excellent. Details of quality assessment are presented in Additional file 2: Table S1.

### Assessment of psychometric properties

The timing of assessments of the validity of PROMs varied across studies and sometimes, within the same study [29]. Data on responsiveness were reported for the WIQ [29], ICQ [20]; VascuQoL [22, 23]; SF-8 [21]; SF-36 [19, 21–23]; EQ-5D [19, 22]; Nottingham Health Profile (NHP) and Sickness Impact Profile-intermittent claudication (SIPic) [22]. Test-retest reliability of PROMs was reported in 8 studies. Follow-up periods varied and ranged from 1-week [19, 20, 27]; 2-week [20, 21, 26] to 1-month intervals [23, 30]. A summary of reported psychometric properties of identified PROMs is presented in Table 5.

**Table 5:** Summary of the psychometric properties of patient-reported outcome measures in patients with peripheral arterial disease

	Internal consistency	Test-retest	Content validity	Construct validity	Responsiveness	Floor/ ceiling	Acceptability
Generic PROMs							
EQ-5D							
Chetter 1997 [19]	0	?	0	−/+	−/+	0	0
Chong 2002 [20]	0	0	0	?	−/+	0	0
Coyne 2003 [26]	0	0	0	−/+	0	0	0
Mazari 2010 [25]	0	0	0	+	−/+	0	0
Mehta 2006 [22]	0	0	0	?	+	0	0
NHP							
Chetter 1997 [19]	0	?	0	−/+	+	−/+	0
POMS							
Treat-Jacobson 2012 [31]	0	0	0	−/+	0	0	0
SF-6D [25]	0	0	0	−/+	+	0.	0
SF-8							
Gulati 2009 [21]	0	?	0	+	−/+	0	0
SF-36							
Chetter 1997 [19]	0	?	0	−/+	−/+	−/+	0
Chong 2002 [20]	0	0	0	+	−/+	0	0
Coyne 2003 [26]	0	0	0	−/+	0	0	0
Gulati 2009 [21]	0	?	0	+	−/+	0	0
Izquierdo-Porrera 2005 [27]	0	0	0	−/+	0	0	0
Mazari 2010 [25]	0	0	0	−/+	+	0	0
Mehta 2006 [22]	0	0	0	+	−/+	0	0
Morgan 2001 [23]	0	0	0	+	−/+	0	0
Smith 2007 [18]	−	+	0	−/+	0	0	?
Spertus 2003 [30]	+	+	0	+	−/+	0	0
Treat-Jacobson 2012 [31]	0	0	0	−/+	0	0	0
Condition-specific PROMs							
AUSVIQUOL							
Smith 2007 [18]	+	+	0	−/+	0	0	?
ICQ							
Chong 2002 [20]	−/+	+	+	−/+	+	?	+
PADQOL							
Treat-Jacobson 2012 [31]	+	0	+	−/+	0	0	0
PAQ							
Spertus 2003 [30]	+	+	+	+	+	0	0
SIPic							
Mehta 2006 [22]	0	0	0	+	−/+	0	0
WIQ							
Chong 2002 [20]	0	0	0	−/+	−/+	0	0
Coyne 2003 [26]	−/+	−/+	0	+	0	0	0
Izquierdo-Porrera 2005 [27]	0	0	0	−/+	0	0	0
McDermott 1998 [28]	0	0	0	−/+	0	0	0
Regensteiner 1990 [29]	0	?	0	−/+	+	0	0
Spertus 2003 [30]	−/+	+	0	+	−/+	0	0
Tew 2013 [24]	0	0	0	−/+	0	0	+
Treat-Jacobson 2012 [31]	0	0	0	−/+	0	0	0
VascuQoL							
Mazari 2010 [25]	0	0	0	+	+	0	0
Mehta 2006 [22]	0	0	0	+	−/+	0	0
Morgan 2001 [23]	+	+	+	+	+	0	0
Psychometric and operational criteria							
0	Not reported (no evaluation completed)						
-	Evidence not in favour						
−/+	Weak evidence in favour						
+	Evidence in favour						
?	Methodology questionable						
N.B. Blank criterion validity excluded from the table.							

NB Criterion validity recorded as zero across studies therefore results are not displayed

Abbreviations: AUSVIQUOL Australian Vascular Quality of Life Index, Q Questionnaire, EQ-5D EuroQol, ICQ Intermittent Claudication Questionnaire, NHP Nottingham Health Profile, PAD peripheral arterial disease, PADQOL PAD Quality of Life Questionnaire, PAQ Peripheral Artery Questionnaire, POMS Profile of Mood States, SF-36 Medical Outcomes Study 36-item Short Form, SF-6D 6-item shortened version of SF-36, SF-8 8-item shortened version of SF-36, SIPic Sickness Impact-Intermittent Claudication, VascuQoL King’s College Hospital’s Vascular Quality of Life instrument, WIQ Walking Impairment Questionnaire

### Generic patient-reported outcome measures

Eleven studies assessed the construct validity of the SF-36. Five studies [20–23, 30] reported good evidence with the remaining presenting mixed evidence. Evidence for the internal consistency of the SF-36 was negative from one study [18] and positive in another study [30]. Only one study [25] reported positive evidence on responsiveness while the six studies [19–23, 30] found mixed evidence. Test-retest reliability was assessed in 4 studies with 2 studies providing evidence in favour of test re-test reliability [18, 30]; one study [19] describing positive evidence on test-retest reliability using simple correlations but providing no information on time interval for the administration of the measures and the remaining study [21] assessing reliability using Spearman correlations instead of intra-class correlation coefficients.

Positive evidence for construct validity and mixed evidence of responsiveness of the SF-8 were reported [21]. One study provided mixed evidence for construct validity and positive evidence for responsiveness of the SF-6D [25]. The quality of study methodology was shown to be good for construct validity, mixed for test-re-test reliability, and poor for responsiveness.

Of the 5 studies evaluating the EQ-5D, one study showed positive evidence for construct validity [25]; 2 studies reported mixed evidence [19, 26] whereas the remaining studies [20, 22] had poor methodologies, subsequently limiting further assessment.

The responsiveness of the NHP was found to be favourable but construct validity and floor/ceiling effects were associated with mixed evidence [19]. For examining construct validity of the Profile of Mood States (POMS), no prior hypotheses of the strength and direction that the POMS would be related to other measures was reported [31]. However, the results presented showed statistically significant correlations with the PADQOL factors [31].

### Condition-specific patient-reported outcome measures

Three papers evaluating the VascuQol provided good evidence for its construct validity and responsiveness [22, 23, 25]. Content validity and internal consistency were found to be positive in the one study [23] with some evidence in favour of the test-re-test reliability [23]. Evidence for internal consistency, test re-test reliability, responsiveness and acceptability were explored in relevant studies relating to the WIQ [20, 24, 26–31]. On the other hand, Spertus et al. [30] reported Cronbach’s alpha of 0.94, indicating a possible overlap with other domains on the measure. Two studies [26, 30] found good evidence for the construct validity of the WIQ; however the others [20, 24, 27–29] reported inconsistent evidence. A single study of exercise therapy [29] found positive evidence for the responsiveness of the scale but mixed evidence was described by two studies [20, 30].

One study reported good evidence on internal consistency and reliability of the AUSVIQOL in patients with PAD [18], but there was mixed evidence for construct validity. Overall, the study’s methodology was rated as fair. Good evidence about the internal consistency, test-retest reliability, construct validity and responsiveness of the PAQ was presented by Spertus et al. [30]. The PAQ was developed after a review of the medical literature, examination of the available measures, focus groups with clinicians and unstructured interviews with patients suggesting positive content validity. However, the methodology of the study was found to be poor. Good evidence was observed for the internal consistency and content validity of the PADQOL in one study [31]. Generally, the reported methodology was rated as good, but construct validity was found to have mixed evidence due to the lack of prior hypotheses [31]. The measurement properties of the ICQ were examined in a study [20] that reported a Cronbach’s alpha of 0.94, indicating high correlation between items. However, positive results were found for the test-retest reliability, content validity and responsiveness. In this study, mixed evidence was found for the construct validity due to a lack of a clear hypothesis. The methodology to assess these criteria was generally good, although the responsiveness received only a fair rating [20]. The SIP_IC_ was evaluated with patients with lifestyle-limiting claudication [22]. Good evidence was found for construct validity and mixed evidence for responsiveness.

Two studies reported the psychometric assessment of modified PROM instruments. These were the modified telephone-administered WIQ [26] and the SF-8, an abridged version of the SF-36 [21]. Both the originally developed telephone-administered WIQ and the modified self-administered version were reported to be valid and reliable for objectively assessing community walking. The authors proposed that self-administration reduced the WIQ completion time, from five minutes to one minute. [26].

## Discussion

Fourteen studies assessing the psychometric properties of 13 newly-developed and existing PROMs in patients with symptomatic PAD, regardless of specific presentation were included in this review. Substantial variations in the reporting of clinical presentation of PAD, management strategies and administration of instruments were noted. Evidence of superiority in the psychometric performance of a single PROM could not be established. This may be a reflection of the differences in patient characteristics and study methodology rather than the appropriateness of the instruments themselves.

Clinicians and researchers have a wide variety of PROMs to consider for patients with PAD. The review included generic PROMs as well as PROMs that covered PAD-related symptoms e.g. VascuQoL (pain); WIQ (walking speed) and PADQoL (symptoms and limitations of function fear and anxiety). Of the generic PROMs evaluated, the SF-36 showed the most complete and positive evidence in favour of use in a PAD population. The domains of the SF-36 provided a broader measure than the PAD-specific PROMs. This instrument included further questioning on the domains of pain and mobility, but also on specific fears. However, related studies were of mixed methodological quality. The review showed that using modified versions of the WIQ and SF-36 provided useful PROMs data in terms of test re-test reliability, construct validity and responsiveness. Nonetheless, adopting these instruments in practice requires more consideration of their appropriateness considering the extent of variation in the available literature. Although the WIQ provides a good condition-specific measure of mobility relevant to IC, it does not include QOL measures relating to PAD, in general. The VascuQol was found to have good internal consistency, test-retest reliability, construct validity and responsiveness as well as good content validity for measuring QOL of patients with PAD.

Several factors may influence the choice of a PROM. Careful consideration is required regarding whether a combination of measures should be recommended for use in symptomatic patients or whether a single PROM covering different aspects of health would be more appropriate for obtaining the patient’s perspective on treatment and general health. Furthermore, patients’ characteristics (stage of PAD, treatment, co-existing conditions) must be carefully considered. Included studies dealt with patients with symptomatic PAD and more research is needed to understand the relevance of using PROMs in those with asymptomatic PAD. This is of particular importance because PAD represents a continuum of clinical presentations. A decision about whether or not to use a single PROM or set of PROMs in practice should be at the discretion of clinicians or researchers. One key area of attention, however, should include the burden of administering a questionnaire (including format, setting, time for completion). In the study by Coyne et al. [26], the authors reported that the modified (self-administered) WIQ was reliable and valid when compared to the version administered by an interviewer over the telephone [26]. However, recent evidence suggests that the number of errors occurring during self-completion of the WIQ was unacceptably high [33, 34] and this will have implications for administering a tool as well as interpreting the findings of the self-completed PROMs. Furthermore, limited or unclear reporting makes inferences about completion time reasonably challenging. Methods for calculating completion time, additional support provided and reading level of participants were often not reported within included studies.

Whereas it is not possible to single out one measure for recommendation, it is evident that condition-specific measures were the only tools with reported content validity related to PAD. Based on the findings of this review, the PAQ and VascuQoL would seem to be appropriate condition-specific tools for predominantly English speakers. The ICQ could be selected as a tool of choice for patients with intermittent claudication, only. Measurements of PROMs must be practical, acceptable and reliable. Therefore, qualitative evidence based on patients’ views and experiences will also be valuable. Additionally, clinical trials which incorporate PROMs as outcome measures may be used to assess the performance of relevant PROMs but this is beyond the scope of this paper. Collectively, such evidence will help in selecting PROMs for use in routine practice.

Clear and complete reporting of validation studies is essential. The quality of reporting in the included studies was often, inadequate or ambiguous. For example, patient selection was not presented in a meaningful way in most studies. Whilst some studies explicitly stated that patients with more severe forms of PAD were excluded, a few studies did not provide information to identify any stratification of the study population. The ankle-brachial index (ABI) cut-offs for selecting patients in included studies were often not reported or varied across studies.

In this review, the methodological quality of the studies was evaluated on the basis of the COSMIN criteria. However, this checklist is time-consuming to apply and although it provides a method for assessing the quality of the studies, it has been criticised for being difficult to apply in a consistent manner [14]. The current review, similar to the study by Morris et al. (2014) [14], also demonstrated that many of the included studies had not reported on how missing information was handled. The approach used for handling missing data is a key criterion for the COSMIN checklist. Subsequently, most of the studies were rated low in terms of quality. Another systematic review of PROMs in patients with IC found that the methodological quality of most studies ranged from poor to fair [35]. Our review supports the findings of the review by Conijn et al. [35] confirming the need for better quality studies of PROMs.

### Strengths and Limitations of the review

Comprehensive and iterative literature searching was undertaken to improve the retrieval of relevant studies. Our efforts improved article retrieval because more than half of included studies were not identifiable as validation studies by titles only. This review identified PROMs for patients with IC and other stages of PAD. It is possible that the differences in clinical states may have influenced the findings of psychometric assessments. Previous reviews have been much more restricted in their scope and limited in the range of sources searched. By broadening the scope of the population of interest, this study has also highlighted the evidence gap regarding validation of PROMs in patients with more severe forms of PAD or more specifically, patients with amputation due to PAD.

In an effort to identify suitable PROMs for patients receiving care for PAD within the NHS in England, we excluded non-English populations or PROMs developed or available in other languages other than English. As a result, potentially informative data, for example, from validation of *non-eligible PROMs* [36–38] was not included in this study. The impact of excluding non-English populations or PROMs in this review is unclear. However, this approach was reasonable because of challenges with linguistic validation and cultural adaptation of outcome measures [9]. Literature searches were updated in January 2015 so more recent relevant studies may have been missed.

### Implications for practice and future research

Due to heterogeneity and methodological quality of studies included in this review, no single PROM can be recommended for use. It is recommended that clinicians and researchers take into account the factors related to the burden of administration, patient characteristics and treatment strategies when selecting appropriate PROMs. Any suitable instrument should aim to cover all relevant domains of interest to patients.

The standardisation of study methodology and reporting must be encouraged with the view to improve interpretation of findings of validation studies. Existing minimum standards for PROMs [39] provides useful guidelines in designing, choosing and validating PROMs. The latter can be used alongside the COSMIN checklist to design and reporting validation studies. The next stage of our research is to complete a qualitative review to obtain patient’s views about factors that significantly affect their daily functioning and QoL whilst living with PAD and a review of PROMs as outcomes in randomised studies. It is anticipated that the evidence created will inform the selection or development of a new tool to obtain PROMs in patients with PAD attending clinics within the NHS, England.

## Conclusions

This review provides an in-depth summary of PROMs evaluated in English-speaking patients with symptomatic PAD. No study provided evidence of a full psychometric evaluation in the patient population of interest. The consideration of diverse factors will help to identify a suitable PROM or combination of measures for clinical and health care decision-making. Additionally, standardised methodologies will help to substantially improve the interpretation of findings from validation studies.

## Additional files

Additional file 1: Search strategies (DOCX 28 kb)
Additional file 2: Table S1. Methodological assessment of quality of each PROM by study using the COSMIN criteria (DOCX 75 kb)

## Abbreviations

AUSVIQUOL: Australian Vascular Quality of Life Index
CLAU-S: Claudication Scale
CLI: Critical limb ischaemia
COSMIN: Consensus-based Standards for the selection of health Measurement INstruments
EACH-Q: Estimation of Ambulatory Capacity by History-Questionnaire
EMBASE: Excerpta Medica dataBASE
ePAQ: Electronic personal assessment questionnaire
EQ-5D: EuroQoL-5D
IC: Intermittent claudication
ICQ: Intermittent Claudication Questionnaire
MeSH: Medical Subject Heading
NHP: Nottingham Health Profile
NHS: National Health Service
NR: Not reported
PAD: Peripheral arterial disease
PADQOL: PAD Quality of Life Questionnaire
PAQ: Peripheral Artery Questionnaire
POMS: Profile of Mood States
PRISMA: Preferred Reporting Items for Systematic Reviews and Meta-Analysis
PROM: Patient-reported outcome measure
PROQOLID: Patient-Reported Outcome and Quality of Life Instruments database
QoL: Quality of life
SF-36: Medical Outcomes Study 36-item short form health survey
SF-6D: 6-item shortened version of SF-36
SF-8D: 8-item shortened version of SF-36
SG: Standard gamble
SIP_ic_: Sickness Impact Profile-Intermittent Claudication
TTO: Time trade-off
VAS: Visual analogue scale
VascuQoL: King’s College Hospital’s Vascular Quality of Life instrument
WIQ: Walking Impairment Questionnaire

## References

[1] Dawson J, Doll H, Fitzpatrick R, Jenkinson C, Carr A (2010). The routine use of patient reported outcome measures in healthcare settings. Br Med J.

[2] Fitzpatrick R, Davey C, Buxton MJ, Jones DR. Evaluating patient-based outcome measures for use in clinical trials. Health technol assess. 1998;2(14):1–74. 9812244

[3] Health, Social Care Information. Finalised Patient Reported Outcome Measures (PROMs) In England: April 2011 to March 2012. 2014. Available at http://www.content.digital.nhs.uk/catalogue/PUB11359/final-proms-eng-apr11-mar12-fin-report-v2.pdf. Accessed 15 Nov 2016.

[4] Regensteiner JG, Hiatt WR (2002). Current medical therapies for patients with peripheral arterial disease: a critical review. Am J Med.

[5] Hiatt WR (2001). Medical treatment of peripheral arterial disease and claudication. N Engl J Med.

[6] Macefield RC, Jacobs M, Korfage IJ, Nicklin J, Whistance RN, Brookes ST, Sprangers MA, Blazeby JM (2014). Developing core outcomes sets: methods for identifying and including patient-reported outcomes (PROs). Trials.

[7] Liberati A, Altman DG, Tetzlaff J, Mulrow C, Gotzsche PC, Ioannidis JP, Clarke M, Devereaux PJ, Kleijnen J, Moher D (2009). The PRISMA statement for reporting systematic reviews and meta-analyses of studies that evaluate healthcare interventions: explanation and elaboration. BMJ.

[8] Jenkinson C, Gibbons E, Fitzpatrick R (2009). A structured review of patient-reported outcome measures in relation to stroke.

[9] Schellingerhout JM, Heymans MW, Verhagen AP, de Vet HC, Koes BW, Terwee CB (2011). Measurement properties of translated versions of neck-specific questionnaires: a systematic review. BMC Med Res Methodol.

[10] Emery MP, Perrier LL, Acquadro C (2005). Patient-reported outcome and quality of life instruments database (PROQOLID): frequently asked questions. Health Qual Life Outcomes.

[11] McKenna SP (2011). Measuring patient-reported outcomes: moving beyond misplaced common sense to hard science. BMC Med.

[12] Mokkink LB, Terwee CB, Patrick DL, Alonso J, Stratford PW, Knol DL, Bouter LM, de Vet HC (2010). The COSMIN checklist for assessing the methodological quality of studies on measurement properties of health status measurement instruments: an international Delphi study. Qual Life Res.

[13] Terwee CB, Mokkink LB, Knol DL, Ostelo RW, Bouter LM, de Vet HC (2012). Rating the methodological quality in systematic reviews of studies on measurement properties: a scoring system for the COSMIN checklist. Qual Life Res.

[14] Morris C, Janssens A, Allard A, Thompson CJ, Shilling V, Tomlinson R, Williams J, Fellowes A, Rogers M, Allen K et al. Informing the NHS Outcomes Framework: evaluating meaningful health outcomes for children with neurodisability using multiple methods including systematic review, qualitative research, Delphi survey and consensus meeting. Health Serv Deliv Res. 2014;2(15). 25642547

[15] Terwee CB, Bot SD, de Boer MR, van der Windt DA, Knol DL, Dekker J, Bouter LM, de Vet HC (2007). Quality criteria were proposed for measurement properties of health status questionnaires. J Clin Epidemiol.

[16] Lamping DL, Schroter S, Marquis P, Marrel A, Duprat-Lomon I, Sagnier PP (2002). The community-acquired pneumonia symptom questionnaire: a new, patient-based outcome measure to evaluate symptoms in patients with community-acquired pneumonia. Chest.

[17] Cohen J (1988). Statistical Power Analysis for the Behavioral Sciences.

[18] Smith MJ, Borchard KL, Hinton E, Scott AR (2007). The Australian Vascular Quality of Life Index (AUSVIQUOL): an improved clinical quality of life tool for peripheral vascular disease. Eur J Vasc Endovasc Surg.

[19] Chetter IC, Spark JI, Dolan P, Scott DJ, Kester RC (1997). Quality of life analysis in patients with lower limb ischaemia: suggestions for European standardisation. Eur J Vasc Endovasc Surg.

[20] Chong PFS, Garratt AM, Golledge J, Greenhalgh RM, Davies AH (2002). The intermittent claudication questionnaire: a patient-assessed condition-specific health outcome measure. J Vasc Surg.

[21] Gulati S, Coughlin PA, Hatfield J, Chetter IC (2009). Quality of life in patients with lower limb ischemia; revised suggestions for analysis. J Vasc Surg.

[22] Mehta T, Venkata SA, Chetter I, McCollum P (2006). Assessing the validity and responsiveness of disease-specific quality of life instruments in intermittent claudication. Eur J Vasc Endovasc Surg.

[23] Morgan MB, Crayford T, Murrin B, Fraser SC (2001). Developing the Vascular Quality of Life Questionnaire: a new disease-specific quality of life measure for use in lower limb ischemia. J Vasc Surg.

[24] Tew G, Copeland R, Le Faucheur A, Gernigon M, Nawaz S, Abraham P (2013). Feasibility and validity of self-reported walking capacity in patients with intermittent claudication. J Vasc Surg.

[25] Mazari FAK, Carradice D, Rahman MN, Khan JA, Mockford K, Mehta T, McCollum PT, Chetter IC (2010). An analysis of relationship between quality of life indices and clinical improvement following intervention in patients with intermittent claudication due to femoropopliteal disease. J Vasc Surg.

[26] Coyne KS, Margolis MK, Gilchrist KA, Grandy SP, Hiatt WR, Ratchford A, Revicki DA, Weintraub WS, Regensteiner JG (2003). Evaluating effects of method of administration on Walking Impairment Questionnaire. J Vasc Surg.

[27] Izquierdo-Porrera AM, Gardner AW, Bradham DD, Montgomery PS, Sorkin JD, Powell CC, Katzel LI (2005). Relationship between objective measures of peripheral arterial disease severity to self-reported quality of life in older adults with intermittent claudication. J Vasc Surg.

[28] McDermott MM, Liu K, Guralnik JM, Martin GJ, Criqui MH, Greenland P (1998). Measurement of walking endurance and walking velocity with questionnaire: validation of the walking impairment questionnaire in men and women with peripheral arterial disease. J Vasc Surg.

[29] Regensteiner JG, Steiner JF, Panzer RJ, Hiatt WR (1990). Evaluation of walking impairment by questionnaire in patients with peripheral arterial disease. Eur J Vasc Endovasc Surg.

[30] Spertus J, Jones P, Poler S, Rocha-Singh K (2004). The peripheral artery questionnaire: a new disease-specific health status measure for patients with peripheral arterial disease. Am Heart J.

[31] Treat-Jacobson D, Lindquist RA, Witt DR, Kirk LN, Schorr EN, Bronas UG, Davey CS, Regensteiner JG (2012). The PADQOL: development and validation of a PAD-specific quality of life questionnaire. Vasc Med.

[32] Turner RR, Quittner AL, Parasuraman BM, Kallich JD, Cleeland CS (2007). Patient-reported outcomes: instrument development and selection issues. Value Health.

[33] Mahe G, Ouedraogo N, Vasseur M, Faligant C, Saidi K, Leftheriotis G, Abraham P (2011). Limitations of self-reported estimates of functional capacity using the Walking Impairment Questionnaire. Eur J Vasc Endovasc Surg.

[34] Mahe G, Ouedraogo N, Marchand J, Vielle B, Picquet J, Leftheriotis G, Abraham P (2011). Self-reported estimation of usual walking speed improves the performance of questionnaires estimating walking capacity in patients with vascular-type claudication. J Vasc Surg.

[35] Conijn AP, Jens S, Terwee CB, Breek JC, Koelemay MJ (2015). Assessing the quality of available patient reported outcome measures for intermittent claudication: a systematic review using the COSMIN checklist. Eur J Vasc Endovasc Surg.

[36] Ouedraogo N, Mahe G, Marchand J, Saidi K, Leftheriotis G, Abraham P, et al. Validation of a new simple questionnaire to “estimate ambulation capacity by history” (EACH) in patients with claudication. J Vasc Surg. 2011;54(1):133–8. 10.1016/j.jvs.2010.11.129 21334169

[37] Spengel FA, Lehert P, Dietze S (1998). A statistical consideration of CLAU-S: a disease specific questionnaire for the assessment of quality of life in patients with intermittent claudication. VASA.

[38] Wann-Hansson C, Hallberg IR, Risberg B, Klevsgard R (2004). A comparison of the Nottingham Health Profile and Short Form 36 Health Survey in patients with chronic lower limb ischaemia in a longitudinal perspective. Health Qual Life Outcomes.

[39] Reeve BB, Wyrwich KW, Wu AW, Terwee CB, Synder CF, Schwartz C, Revicki DD, Monipour CM, McLeod LD, Lyons JC (2013). ISOQOL recommends minimum standards for patient-reported outcome measures used in patient-centered outcomes and comparative effectiveness research. Qual Life Res.

