# Supplementary material for: Patient-reported outcome measures in patients with peripheral arterial disease: a systematic review of psychometric properties
Source: Health Qual Life Outcomes. 2016 Nov 24;14:161. doi: 10.1186/s12955-016-0563-y (PMC5121983; doi:10.1186/s12955-016-0563-y)
Supplement: Additional file 1: — Search strategies (DOCX 28 kb) [file 12955_2016_563_MOESM1_ESM.docx]

**Additional file 1: Search strategies**

Search One

Database: Ovid MEDLINE(R) In-Process & Other Non-Indexed Citations and Ovid MEDLINE(R) <1946 to Present>

Search Strategy:

--------------------------------------------------------------------------------

1 Peripheral Arterial Disease/

2 exp Peripheral Vascular Diseases/

3 Intermittent Claudication/

4 Amputation/

5 (Peripheral arterial disease$ or peripheral vascular disease$).tw.

6 intermittent claudication.tw.

7 critical limb isch?emia.tw.

8 rest pain.tw.

9 (amputation adj5 (lower limb or lower-limb)).tw.

10 (atherosclero$ and (PAD or PVD)).tw.

11 (arterial disease$ and (PAD or PVD)).tw.

12 arterial occlusive disease.tw.

13 arterial insufficiency.tw.

14 lower limb isch?emia.tw.

15 lower extremity isch?emia.tw.

16 (peripheral and lower extremity).tw.

17 (lower limb$ adj2 (isch?emi$ or arter$ or vein$ or vessel$ or vascular or occlusive)).tw.

18 (lower extremit$ adj2 (isch?emi$ or arter$ or vein$ or vessel$ or vascular or occlusive)).tw.

19 (leg adj2 (isch?emi$ or arter$ or vein$ or vessel$ or vascular or occlusive)).tw.

20 or/1-19

21 (patient reported outcome$ or patient-reported outcome$).tw.

22 (prom or proms).tw.

23 (disease reported outcome$ or disease-reported outcome$).tw.

24 21 or 22 or 23

25 "Quality of Life"/

26 "Outcome and Process Assessment (Health Care)"/

27 "Outcome Assessment (Health Care)"/

28 quality of life.tw.

29 qol.tw.

30 outcome measure$.tw.

31 health outcome$.tw.

32 or/25-31

33 (patient adj20 report$).tw.

34 32 and 33

35 24 or 34

36 (intermittent claudication questionnaire or ICQ or walking impairment questionnaire or claudication outcome measures questionnaire or COM).tw.

37 nottingham health profile.tw.

38 36 or 37

39 health related quality of life.tw.

40 health related qol.tw.

41 health related ql.tw.

42 hrqol.tw.

43 hql.tw.

44 health state utilit$.tw.

45 hsuv$.tw.

46 (euroqol or euro qol or eq5d or eq 5d).tw.

47 (sf6d or sf 6d or sf 6 dimension$ or sf six dimension$ or shortform 6d or shortform six dimension$ or short form 6d or short form 6 dimension$ or short form six dimension$).tw.

48 (sf12 or sf 12 or short form 12 or shortform 12 or sf twelve or sftwelve or shortform twelve or short form twelve).tw.

49 (sf36 or sf 36 or short form 36 or shortform 36 or sf thirtysix or sf thirty six or shortform thirtysix or shortform thirty six or short form thirtysix or short form thirty six).tw.

50 (item adj3 short form).tw.

51 (item adj3 shortform).tw.

52 medical outcomes survey.tw.

53 medical outcomes study.tw.

54 mos.tw.

55 psychological general wellbeing index.tw.

56 psychological general well being index.tw.

57 pgwb$.tw.

58 health utilit$.tw.

59 hui$.tw.

60 quality of wellbeing.tw.

61 quality of well being.tw.

62 qwb$.tw.

63 rosser.tw.

64 trade off$.tw.

65 standard gamble$.tw.

66 tto$.tw.

67 qaly$.tw.

68 quality adjusted life year$.tw.

69 quality-adjusted life years/

70 hye$.tw.

71 health$ year$ equivalent$.tw.

72 disutilit$.tw.

73 disbenefit$.tw.

74 "Quality of Life"/

75 "Outcome Assessment (Health Care)"/

76 quality of life.tw.

77 74 or 75 or 76

78 (preference based or utilit$ or generic preference).tw.

79 77 and 78

80 (preference$ adj2 (elicit$ or patient$ or population$ or measure$ or based or cost$)).tw.

81 (utilit$ adj2 (elicit$ or patient$ or population$ or measure$ or based or cost$)).tw.

82 or/39-73

83 79 or 80 or 81 or 82

84 35 or 38 or 83

85 20 and 84

Search Two

Database: Ovid MEDLINE(R) In-Process & Other Non-Indexed Citations and Ovid MEDLINE(R) <1946 to Present>

Search Strategy:

--------------------------------------------------------------------------------

1 exp Peripheral Vascular Diseases/

2 Intermittent Claudication/

3 Amputation/

4 (Peripheral arterial disease$ or peripheral vascular disease$).tw.

5 intermittent claudication.tw.

6 critical limb isch?emia.tw.

7 rest pain.tw.

8 (amputation adj5 (lower limb or lower-limb)).tw.

9 (atherosclero$ and (PAD or PVD)).tw.

10 (arterial disease$ and (PAD or PVD)).tw.

11 arterial occlusive disease.tw.

12 arterial insufficiency.tw.

13 lower limb isch?emia.tw.

14 lower extremity isch?emia.tw.

15 (peripheral and lower extremity).tw.

16 (lower limb$ adj2 (isch?emi$ or arter$ or vein$ or vessel$ or vascular or occlusive)).tw.

17 (lower extremit$ adj2 (isch?emi$ or arter$ or vein$ or vessel$ or vascular or occlusive)).tw.

18 (leg adj2 (isch?emi$ or arter$ or vein$ or vessel$ or vascular or occlusive)).tw.

19 or/1-18

20 Estimating Ambulation Capacity by History-Questionnaire.mp.

21 EACH-Q.mp.

22 Positive Attitude Toward Physical Activities Questionnaire.mp.

23 Positive Attitude Toward Physical Exertion Questionnaire.mp.

24 Prosthetics Evaluation Questionnaire.mp.

25 PEQ.mp.

26 Peripheral Artery Questionnaire.mp.

27 PAQ.mp.

28 Cardiff Wound Impact Schedule.mp.

29 CWIS.mp.

30 PAOD Physical Activity Recall.mp.

31 Community-based walking ability.mp.

32 Rand-36 Physical Functioning subscale.mp.

33 Prosthetic Evaluation Questionnaire.mp.

34 "Orthotics and Prosthetics Users Survey".mp.

35 OPUS.mp.

36 Amputee Mobility Predictor.mp.

37 Satisfaction with Prosthesis Questionnaire.mp.

38 SAT-PRO.mp.

39 Rose questionnaire.mp.

40 Australian Vascular Quality of Life Index.mp.

41 AUSVIQUOL.mp.

42 "Disease-specific Questionnaire for Quality of Life in Patients with Peripheral Arterial Occlusive Disease in the Stage of Critical Ischemia".mp.

43 FLeQKI.mp.

44 Claudication Scale questionnaire.mp.

45 CLAU-S.mp.

46 San Diego Claudication Questionnaire.mp.

47 WHO Intermittent claudication questionnaire.mp.

48 Intermittent Claudication Questionnaire.mp.

49 ICQ.mp.

50 Charing Cross Claudication Questionnaire.mp.

51 CCCQ.mp.

52 Claudication Scale.mp.

53 Houghton scale.mp.

54 PAVK-86.mp.

55 Amputee Body Image Scale.mp.

56 ABIS.mp.

57 Vascular Quality of life.mp.

58 VAS-Qol.mp.

59 Edinburgh intermittent claudication questionnaire.mp.

60 EICQ.mp.

61 PAD Quality of life questionnaire.mp.

62 PADQOL.mp.

63 Vascular Quality of life.mp.

64 VascuQol.mp.

65 "Centre for Disease Control and Prevention Health-Related Quality of Life 4 question set".mp.

66 Sickness Impact Profile - Intermittent Claudication.mp.

67 SIPIC.mp.

68 Modified WHO-Edinburgh Claudication Questionnaire.mp.

69 Patient Health Questionnaire.mp.

70 Cumulative Illness Rating Scale.mp.

71 CIRS.mp.

72 Nottingham Health Profile.mp.

73 NHP.mp.

74 Assessment of Quality of Life.mp.

75 AQOL.mp.

76 15D Health-related QoL instrument.mp.

77 6-item Brief Social Support Questionnaire.mp.

78 Geriatric Depression Scale.mp.

79 GDS.mp.

80 Self-reported Life Satisfaction score.mp.

81 Self-reported LS score.mp.

82 "Short-Form Health Survey questionnaire adapted for the veteran population".mp.

83 (SF-36V or SF36V).mp.

84 (short form 36V or short-form 36V).mp.

85 (sf6d or sf 6d or sf 6 dimension$ or sf six dimension$ or shortform 6d or shortform six dimension$ or short form 6d or short form 6 dimension$ or short form six dimension$).mp.

86 (sf36 or sf 36 or short form 36 or shortform 36 or sf thirtysix or sf thirty six or shortform thirtysix or shortform thirty six or short form thirtysix or short form thirty six).mp.

87 (sf8 or sf 8 or short form 8 or shortform 8 or sf eight or sfeight or shortform eight or short form eight).mp.

88 (sf12 or sf 12 or short form 12 or shortform 12 or sf twelve or sftwelve or shortform twelve or short form twelve).mp.

89 (sf20 or sf 20 or short form 20 or shortform 20 or sf twenty or sftwenty or shortform twenty or short form twenty).mp.

90 MOS-SS questionnaire for social support.mp.

91 Zung-SDS.mp.

92 McGill Pain Questionnaire.mp. or Pain Measurement/

93 Functional Limitations Profile.mp.

94 Beck Depression Inventory.mp.

95 Beck Anxiety Inventory.mp.

96 Verbal rating scale.mp.

97 Sickness Impact Profile.mp. or Sickness Impact Profile/

98 SIP.mp.

99 (Hospital Anxiety and Depression Scale).mp.

100 HADS.mp.

101 Global Mood Scale.mp.

102 Patient Generated Index.mp.

103 Standard gamble$.mp.

104 rosser.tw.

105 Quality of Well Being scale.mp.

106 Rand-36 DLV.mp.

107 "Nottingham Health Profile index of Depression".mp.

108 NHPD.mp.

109 (euroqol or euro qol or eq5d or eq 5d).mp.

110 Roland-Morris Disability Questionnaire.mp.

111 Health Utilities Index-Mark III.mp.

112 HUI-III.mp.

113 WHOQOL-100.mp.

114 World Health Organization Quality of Life-BREF.mp.

115 WHOQOL-BREF.mp.

116 WHO questionnaire.mp.

117 Profile of Mood States.mp.

118 POMS.mp.

119 Mental component scale.mp.

120 MCS.mp.

121 Assessment of Quality of Life.mp.

122 (AQoL-4D or AQoL-8D).mp.

123 (walking impairment questionnaire or claudication outcome measures questionnaire or COM).mp.

124 nottingham health profile.mp.

125 OR/20-124

126 19 and 125

127 instrumentation.sh.

128 methods.sh.

129 Validation Studies.pt.

130 Comparative Study.pt.

131 Psychometrics/

132 psychometr*.ti,ab.

133 clinimetr*.tw.

134 clinometr*.tw.

135 "Outcome Assessment (Health Care)"/

136 outcome assessment.ti,ab.

137 outcome measure*.tw.

138 Observer Variation/

139 observer variation.ti,ab.

140 Health Status Indicators/

141 "Reproducibility of Results"/

142 reproducib*.ti,ab.

143 Discriminant Analysis/

144 reliab*.ti,ab.

145 unreliab*.ti,ab.

146 valid*.ti,ab.

147 coefficient.ti,ab.

148 homogeneity.ti,ab.

149 homogeneous.ti,ab.

150 "internal consistency".ti,ab.

151 OR/127- 150

152 cronbach*.ti,ab.

153 (alpha or alphas).ti,ab.

154 152 and 153

155 item.ti,ab.

156 (correlation* or selection* or reduction*).ti,ab.

157 155 and 156

158 (agreement or precision or imprecision or "precise values" or "test–retest").ti,ab.

159 (test and retest).ti,ab.

160 reliab*.ti,ab.

161 (test or retest).ti,ab.

162 160 and 161

163 154 or 157 or 158 or 159 or 162

164 (stability or interrater or inter-rater or intrarater or intra-rater or intertester or inter-tester or intratester or intra-tester or interobserver or inter-observer or intraobserver or intra-observer).ti,ab.

165 (intertechnician or inter-technician or intratechnician or intra-technician or interexaminer or inter-examiner or intraexaminer or intra-examiner or interassay or inter-assay or intraassay or intra-assay or interindividual or inter-individual or intraindividual or intra-individual or interparticipant or inter-participant or intraparticipant or intra-participant).ti,ab.

166 (kappa or "kappa’s" or kappas or repeatab*).ti,ab.

167 repeatab*.ti,ab.

168 164 or 165 or 166 or 167

169 (replicab* or repeated).ti,ab.

170 (measure or measures or findings or result or results or test or tests).ti,ab.

171 169 and 170

172 (generaliza* or generalisa* or concordance).ti,ab.

173 (intraclass and correlation*).ti,ab.

174 (discriminative or "known group" or factor analysis or factor analyses or dimension* or subscale*).ti,ab.

175 (multitrait and scaling and (analysis or analyses)).ti,ab.

176 171 or 172 or 173 or 174 or 175

177 (item discriminant or interscale correlation* or error or errors or "individual variability").ti,ab.

178 (variability and (analysis or values)).ti,ab.

179 (uncertainty and (measurement or measuring)).ti,ab.

180 ("standard error of measurement" or sensitiv* or responsive*).ti,ab.

181 ((minimal or minimally or clinical or clinically) and (important or significant or detectable) and (change or difference)).ti,ab.

182 (small* and (real or detectable) and (change or difference)).ti,ab.

183 (meaningful change or "ceiling effect" or "floor effect" or "Item response model" or IRT or Rasch or "Differential item functioning" or DIF or "computer adaptive testing" or "item bank" or "cross-cultural equivalence").ti,ab.

184 177 or 178 or 179 or 180 or 181 or 182 or 183

185 151 or 163 or 168 or 176 or 184

186 126 and 185
