# Supplementary material for: Patient-reported outcome measures in patients with peripheral arterial disease: a systematic review of psychometric properties
Source: Health Qual Life Outcomes. 2016 Nov 24;14:161. doi: 10.1186/s12955-016-0563-y (PMC5121983; doi:10.1186/s12955-016-0563-y)
Supplement: Additional file 2: Table S1. — Methodological assessment of quality of each PROM by study using the COSMIN criteria (DOCX 75 kb) [file 12955_2016_563_MOESM2_ESM.docx]

**Revised: 15 November 2016**

**Additional file 2: Table S1 Methodological assessment of quality of each PROM by study using the COSMIN criteria**

| Criteria | Internal Consistency | Reliability | Measurement error | Content validity | Structural validity | Hypothesis testing | Responsive-ness |
| --- | --- | --- | --- | --- | --- | --- | --- |
| *Generic PROMs* | | | | | | | |
| ***EQ-5D*** |  |  |  |  |  |  |  |
| Chetter 1997[19] | . | Poor | Poor | . | . | Poor | Poor |
| Chong 2002[20] | Good | . | . | . | . | Poor | Poor |
| Coyne 2003[26] | . | . | . | . | . | Poor | . |
| Mehta 2006[22] | . | . | . | . | . | Poor | Fair |
| Mazari 2010 [25] | . | . | . | . | . | Poor | Good |
| ***SF-36*** |  |  |  |  |  |  |  |
| Chetter 1997[19] | . | Poor | Poor | . | . | Poor | Poor |
| Chong 2002[20] | Good | . | . | . | . | Poor | Poor |
| Coyne 2003[26] | . | . | . | . | . | Poor | . |
| Gulati 2009[21] | . | Fair | Fair | . | . | Good | Poor |
| Izquierdo-Porrera 2005[27] | . | . | . | . | . | Poor | . |
| Mehta 2006[22] | . | . | . | . | . | Fair | Fair |
| Morgan 2001[23] | . | . | . | . | . | Fair | Fair |
| Smith 2007[18] | Poor | Poor | Poor | . | . | Fair | . |
| Spertus 2003[30] | Poor | Fair | Fair | . | . | Fair | Fair |
| Treat-Jacobson 2012[31] | . | . | . | . | . | Good | . |
| ***SF-6D*** |  |  |  |  |  |  |  |
| Mazari 2010[25] | . | . | . | . | . | Poor | Good |
| ***SF-8*** |  |  |  |  |  |  |  |
| Gulati 2009[21] | . | Fair | Fair | . | . | Good | Poor |
| ***NHP*** |  |  |  |  |  |  |  |
| Chetter 1997[19] | . | Poor | Poor | . | . | Poor | Poor |
| ***POMS*** |  |  |  |  |  |  |  |
| Treat-Jacobson 2012[31] | . | . | . | . | . | Good | . |
| *Condition specific PROMs* | |  |  |  |  |  |  |
| ***AUSVIQUOL*** |  |  |  |  |  |  |  |
| Smith 2007[18] | Poor | Poor | Poor | . | . | Fair | . |
| Mehta 2006[22] | . | . | . | . | . | Fair | Fair |
| ***ICQ*** |  |  |  |  |  |  |  |
| Chong 2002[20] | Good | Good | Good | Fair | Good | Poor | Poor |
| ***PADQOL*** |  |  |  |  |  |  |  |
| Treat-Jacobson 2012[31] | Good | . | . | Good | Good | Good | . |
| ***PAQ*** |  |  |  |  |  |  |  |
| Spertus 2003[30] | Poor | Fair | Fair | . | . | Fair | Fair |
| ***SIPic*** |  |  |  |  |  |  |  |
| Mehta 2006[22] | . | . | . | . | . | Fair | Fair |
| ***VASCUQOL*** |  |  |  |  |  |  |  |
| Mehta 2006[22] | . | . | . | . | . | Fair | Fair |
| Morgan 2001[23] | Poor | Poor | Poor | Good | . | Fair | Fair |
| Mazari 2010 [25] |  |  |  |  |  |  |  |
| ***WIQ*** |  |  |  |  |  |  |  |
| Chong 2002[20] | Good | Good | Good | Fair | Good | Poor | Poor |
| Coyne 2003[26] | Poor | Fair | Fair | . | . | Poor | . |
| Izquierdo-Porrera 2005[27] | . | . | . | . | . | Poor | . |
| McDermott 1998[28] | . | . | . | . | . | Fair | . |
| Regensteiner 1990[29] | . | Poor | . | . | . | Poor | Poor |
| Spertus 2003[30] | Poor | Fair | Fair | . | . | Fair | Fair |
| Tew 2013[24] | . | . | . | . | . | Fair | . |
| Treat-Jacobson 2012[31] | . | . | . | . | . | Good | . |
| Abbreviations: AUSVIQUOL, Australian Vascular Quality of Life Index;; ECQ, EQ-5D, EuroQol; ICQ, Intermittent Claudication Questionnaire; NHP, Nottingham Health Profile; PAD, peripheral arterial disease; PADQOL, PAD Quality of Life Questionnaire; PAQ, Peripheral Artery Questionnaire, POMS, Profile of Mood States; SF-36, Medical Outcomes Study 36-item Short Form; SF-6D, 6-item shortened version of SF-36; SF-8, 8-item shortened version of SF-36; SIPic, Sickness Impact Profile – Intermittent Claudication; VASCuQoL, King’s College Hospital’s Vascular Quality of Life instrument; WIQ, Walking Impairment Questionnaire  NB: cross cultural validity and criterion validity variables excluded from the table. | | | | | | | |
